# Cell-autonomous co-stimulatory function of membrane-bound CD100 promotes activation and differentiation of HBcAg-specific CD8^+^ T cells

**DOI:** 10.3389/fimmu.2026.1826036

**Published:** 2026-06-03

**Authors:** Mengxiao Zhao, Liwei Chen, Yuhang Chen, Lu Wang, Xuecheng Yang, Kathrin Sutter, Mengji Lu, Jieliang Chen, Xin Zheng, Dongliang Yang, Liang Wang, Jia Liu

**Affiliations:** 1 Department of Infectious Diseases, Union Hospital, Tongji Medical College, Huazhong University of Science and Technology, Wuhan, China; 2 Institute of Infectious Diseases and Immunity, Union Hospital, Tongji Medical College, Huazhong University of Science and Technology, Wuhan, China; 3 Institute for Virology, University Hospital Essen, University of Duisburg-Essen, Essen, Germany; 4 School of Basic Medical Sciences, Shanghai Medical College, Fudan University, Shanghai, China; 5 Department of Urology, Union Hospital, Tongji Medical College, Huazhong University of Science and Technology, Wuhan, China

**Keywords:** CD8^+^ T cells, costimulatory molecular, hepatitis B virus, JAK-STAT, membrane-bound CD100, NF-κB, PI3K-Akt-mTOR, transfer

## Abstract

**Background:**

CD100/SEMA4D functions as a critical co-stimulatory molecule in T cell responses, existing in both membrane-bound (mCD100) and soluble (sCD100) forms. Previous studies have shown that CD100 enhances CD8^+^ T cell responses indirectly by promoting the maturation and activation of antigen-presenting cells, thereby facilitating viral clearance. However, whether mCD100, highly expressed on resting T cells, can also directly regulate CD8^+^ T cell function upon ligand engagement remains unclear.

**Methods:**

We examined the impact of CD100 deficiency on the functional phenotype and differentiation of HBcAg-specific CD8^+^ T cells using separate and co-culture systems *in vitro*. Furthermore, through adoptive transfer of CD100 knockout HBV Cor93 TCR-transgenic (CD100KO C93-TCRtg) CD8^+^ T cells, we investigated the early impact of mCD100 deficiency on the development of HBcAg-specific CD8^+^ T cell responses during HBV infection.

**Results:**

The absence of mCD100 expression significantly decrease HBcAg-specific CD8^+^ T-cell proliferation, activation, effector cytokine production, and expression of Granzyme B and Eomes upon antigen stimulation *in vitro*. In the acute self-resolving models, the early proliferation of transferred CD100KO C93-TCRtg CD8^+^ T cells were impaired compared to their WT counterparts following HBV challenge. Transcriptomic data revealed that significant downregulation of gene sets associated with the PI3K-Akt, mTOR, NF-κB, and JAK-STAT signaling pathways in CD100KO CD8^+^ T cells.

**Conclusions:**

Membrane CD100 expressed on resting HBcAg-specific CD8^+^ T cells may function as a co-stimulatory molecule by providing a direct stimulatory signal for the early proliferation and effector differentiation of T cells. The observed effects of mCD100 are associated with the PI3K-Akt-mTOR, JAK-STAT and NF-κB signaling pathways.

## 1 Introduction

Hepatitis B virus (HBV) infection remains a major global public health burden. Approximately 254 million are chronically infected, causing more than 1.1 million annual deaths (1, 2). The clearance of HBV relies largely on a robust T cell response, which is in a state of dysfunction during chronic infection. Recent evidence has shown that multiple co−signaling molecules are involved in regulating the function and differentiation of HBV−specific CD8^+^ T cells, thereby influencing their fate (3).

CD100 (also called SEMA4D) is a transmembrane glycoprotein belonging to the semaphorin family, initially identified as a regulator of neural development and synapse formation (4–7). In lymphoid tissues, mCD100 is highly expressed on the surface of resting T cells, whereas its expression is low on antigen−presenting cells (APCs) such as B cells and dendritic cells (DCs) (5, 8, 9). Upon activation, mCD100 expression is upregulated and can be cleaved by various matrix metalloproteinases (MMPs) into sCD100, both of which play important roles in immune regulation. Through binding to its receptor CD72, CD100 participates in regulating DC maturation, B cell proliferation and antibody production, as well as T−cell activation (5, 6, 10). DCs from CD100-deficient mice exhibit reduced immunogenicity, with impaired expression of costimulatory molecules and defective IL−12 secretion upon CD40 stimulation. Exogenous sCD100 treatment can restore and even enhance DCs function (11). Furthermore, antigen−specific T cell proliferation is impaired in these mice, a defect that can be reversed by recombinant sCD100 treatment (12).

Our previous work demonstrated that serum sCD100 levels were significantly elevated upon acute−resolving HBV challenge, whereas CHB patients showed markedly lower sCD100 levels in PBMC compared with healthy controls. *In vitro* and *in vivo* experiments further confirmed that sCD100 treatment notably enhanced the expression of costimulatory molecules (CD80 and CD86) and IL−12 secretion in APCs such as DCs. In chronic HBV replication mouse models, treatment with recombinant sCD100 significantly enhanced intrahepatic HBV−specific CD8^+^ T cell responses and promoted viral clearance, whereas blocking the interaction between CD100 and CD72 weakened this response and delayed viral clearance (13). Thus, sCD100 indirectly regulates HBV-specific CD8^+^ T cells via APC activation.

Although prior studies from our group and others have established that CD100 can serve as an indirect co-stimulatory molecule by activating APCs, a critical unresolved question is whether mCD100 which is highly expressed on resting T cells can directly regulate CD8^+^ T cell responses during HBV infection. To address this issue, we used CD100KO C93-TCRtg mice, combined with *in vitro* culture systems and *in vivo* adoptive transfer experiments, to systematically investigate the direct regulatory role of mCD100 in the activation and differentiation of HBV−specific CD8^+^ T cells upon HBV exposure. This work aims to further complement and deepen our understanding of the functional mechanisms of CD100 in antiviral immunity.

## 2 Materials and methods

### 2.1 Mice

HBV Cor93 TCR-transgenic (C93-TCRtg) mice were purchased from the Jackson Laboratory. This strain (#027509) has a CD8^+^ T cell repertoire that is specific for the K^b^-restricted COR93 epitope [hepatitis B virus core/nucleocapsid protein residues 93–100 (MGLKFRQL)], as well as the CD45.1 allele. More detailed information could be found at https://www.jax.org/strain/027509. CD100KO mice on a C57BL/6J genetic background were kindly provided by Professor Wei Li Huashan Hospital, Fudan University. The mice were originally produced by Shi et al (12). CD45.1 congenic mice were a gift from Professor Ulf Dittmer (University of Duisburg-Essen, Germany). CD100KO C93-TCRtg mice were generated by crossing C93-TCRtg mice with CD100KO mice. Male C57BL/6J mice, 6–8 weeks old, were purchased from Hunan Slack King Laboratory Animal Co., Ltd. All experimental animals used in this study were bred and kept under specific pathogen-free (SPF) conditions at the Animal Care Center of Tongji Medical College, Huazhong University of Science and Technology.

### 2.2 Anesthesia and euthanasia

All procedures involving animals were performed under inhalation anesthesia to minimize suffering. For retro-orbital blood collection, mice were placed in an anesthesia induction chamber connected to a vaporizer and anesthetized with isoflurane at a concentration of 3–4% in oxygen until the loss of righting reflex was achieved. At the experimental endpoints, mice were humanely euthanized by overdose of isoflurane inhalation (5%) followed by cervical dislocation as a secondary physical method to ensure death.

### 2.3 Acute resolving HBV replicating mouse model

A replication-competent HBV clone pSM2 harboring a head-to-tail tandem dimeric HBV genome (GenBank accession number: V01460) was provided by Dr. Hans Will (Heinrich Pette-Institute, Hamburg, Germany) and used previously in our lab (14). Six- to eight-week-old male mice received a hydrodynamic tail vein injection of 10 µg pSM2 in 0.1 ml/g saline within 5 seconds.

### 2.4 Cell isolation

A single-cell suspension of splenocytes was obtained by grinding spleens through a 70-μm filter. Mouse liver-infiltrating lymphocytes (LILs) were isolated by Percoll density gradient centrifugation after portal vein perfusion with PBS, as described previously (15). Briefly, mouse livers were perfused with 10 ml of PBS immediately after sacrifice. The livers were then homogenized, and the cell pellet was collected. The pellet was resuspended in 40% Percoll and centrifuged at 1000 × g without interruption. After removing the top layer containing debris and hepatocytes, the LILs in the pellet were collected, washed, and prepared for further analysis. For splenocytes preparation, a single-cell suspension was obtained by grinding spleens through a 70-μm filter, followed by red blood cell lysis. After two washes, the splenocyte pellet was collected for further analysis.

### 2.5 *In vitro* stimulation of splenocytes

Freshly isolated splenocytes (SPs) from WT and CD100KO C93-TCRtg mice were stimulated with 10μg/ml HBV Core93–100 peptide (MGLKFRQL) and 1μg/ml anti-CD28 antibody (eBioscience) at 37 °C with 5% CO_2_ for 96 hours. Cells were collected at predetermined time points for further analysis.

### 2.6 Cell sorting and adoptive transfer

CD8^+^ T cells were isolated and purified from mouse splenocytes using a CD8^+^ T Cell Isolation Kit (Miltenyi Biotec). 20,000 purified cells were adoptively transferred into recipient mice via tail vein injection.

### 2.7 Detection of serological HBV infection markers

Serum levels of hepatitis B surface antigen (HBsAg) and hepatitis B e antigen (HBeAg) were measured using enzyme-linked immunosorbent assay (ELISA) kits (KHB, Shanghai, China) according to the manufacturer’s instructions. Serum samples were diluted tenfold with phosphate-buffered saline (PBS) prior to analysis.

### 2.8 Detection of HBV-specific T cells

Freshly isolated LILs or splenocytes were first incubated with anti-CD16/32 antibody (eBioscience) to block Fc receptors, followed by staining with HBV core tetramer (MGLKFRQL)-APC (Institute of Medical Biology) at 4 °C for 60 minutes. After washing, cells were stained with anti-CD8 antibody and a fixable viability dye (FVD, eBioscience), and analyzed by flow cytometry.

### 2.9 Flow cytometry

Cell surface staining and intracellular staining were performed as previously described (16). For surface staining, cells were incubated with the desired antibodies for 30 minutes in the dark at 4 °C. For intracellular staining, cells were fixed and permeabilized using the Intracellular Fixation & Permeabilization Buffer Set (eBioscience) for 30 minutes, followed by antibody staining. For staining of Ki67, Eomes, and T-bet, cells were fixed and permeabilized using the Foxp3/Transcription Factor Staining Buffer Set for 45 minutes at room temperature before staining with the desired antibody combinations. The monoclonal antibodies used are listed in **Supplementary Table 1**. Data were acquired on a FACS Canto II flow cytometer (BD Biosciences) and analyzed using FlowJo v10 software (TreeStar, USA). Cell debris and dead cells were excluded from analysis based on forward/side scatter signals and FVD staining. Absolute cell counts were calculated by multiplying the total viable cell count by the percentage of the relevant cell subset obtained from flow cytometry.

### 2.10 Statistical analysis

RNA sequencing and transcriptomic data analysis were performed by Shanghai Xuran Biotechnology Co., Ltd. Enrichment Scatter Plot, Heatmap Plot and Enrichment Chord Plot were performed using the OmicStudio tools at https://www.omicstudio.cn/tool.

Statistical analyses were performed using GraphPad Prism 9 software. The differences between two groups were evaluated using unpaired *t* test. Data are presented as the mean ± standard error (SEM) and differences were considered statistically significant at *p* < 0.05. Significance is denoted with asterisks (*p < 0.05, **p < 0.01, ***p < 0.001).

## 3 Results

### 3.1 CD100 deficiency impairs proliferation, activation, and effector function of HBcAg-specific CD8^+^ T cells upon antigen stimulation *in vitro*

**Figure 1:**
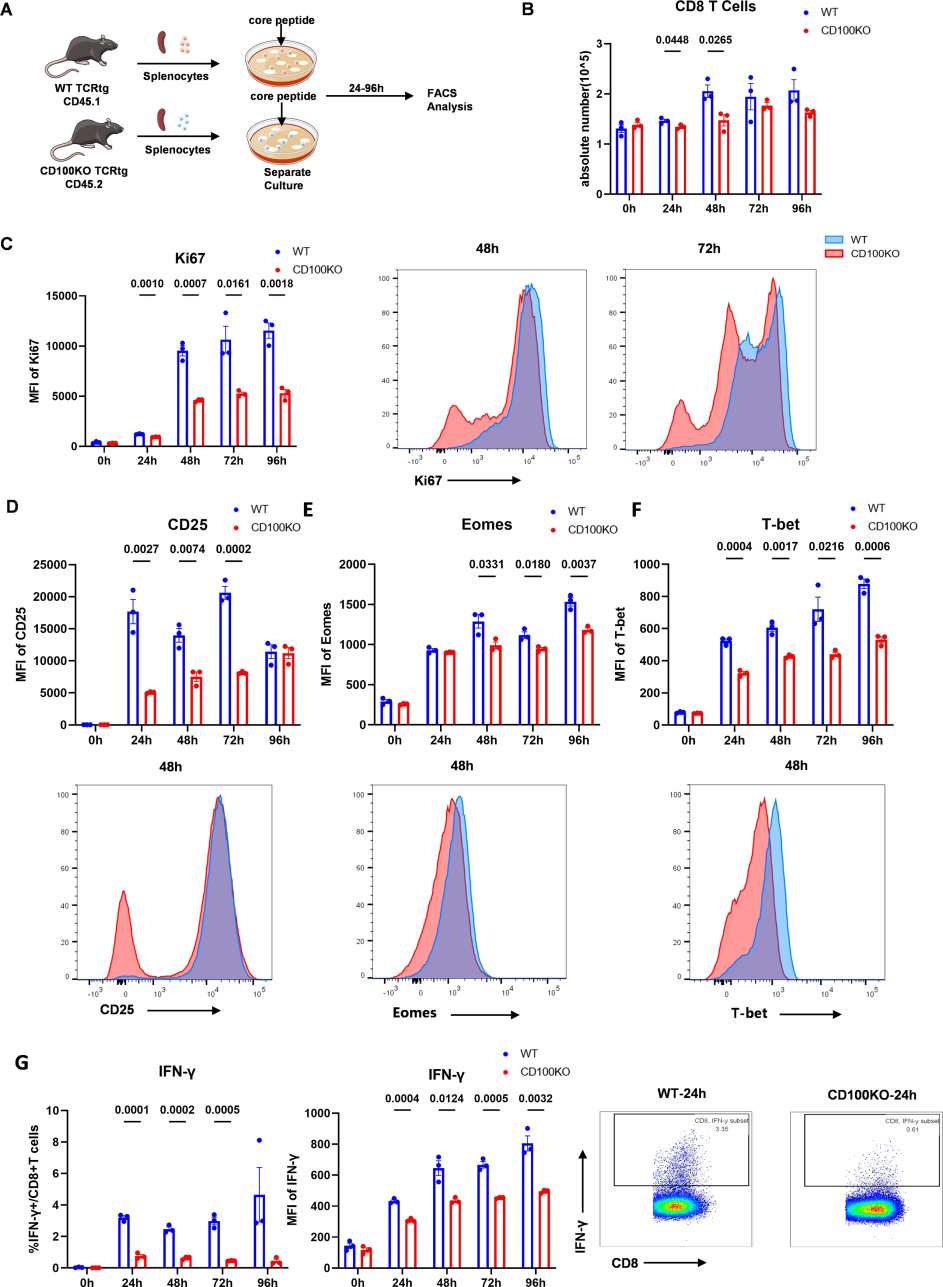
CD100 deficiency impairs proliferation, activation, and effector function of HBcAg-specific CD8^+^ T cells upon antigen stimulation *in vitro*. **(A)** Experimental scheme: Splenocytes from WT C93-TCR-transgenic and CD100KO C93-TCR-transgenic mice were stimulated with Cor93 peptide for 96 hours. **(B)** The absolute number of CD8^+^ T cells at each time point are shown. **(C–F)** Expression levels of Ki67, CD25, Eomes and T-bet in CD8^+^ T cells were analyzed by flow cytometry. **(G)** Frequency and mean fluorescence intensity (MFI) of IFN-γ in CD8^+^ T cells are displayed. Data are depicted as arithmetic means ± SEM. Differences between two groups were analyzed using unpaired Student’s t tests. KO, knockout.

To investigate the impact of CD100 deficiency on the function of HBcAg-specific CD8^+^ T cells, we isolated splenocytes from WT and CD100KO C93-TCRtg mice and stimulated them *in vitro* with the Cor93–100 peptide for 96 hours (**Figure 1A**; **Supplementary Figure 1A**). At 24 and 48 hours after antigen stimulation, the absolute number of CD100KO HBcAg-specific CD8^+^ T cells was significantly lower than that in the WT group (**Figure 1B****).** Consistent with this, the expression level of the proliferation marker Ki67 in CD100KO CD8^+^ T cells was also markedly reduced compared to WT cells (**Figure 1C**; **Supplementary Figure 1B**). At 24 hours post-stimulation, most HBcAg-specific CD8^+^ T cells were activated and expressed CD25 (**Supplementary Figure 1C**); however, CD100 deficiency led to a significant decrease in the MFI of CD25 (**Figure 1D**). We further observed that the expression of the transcription factors Eomes and T-bet was significantly downregulated in CD100KO CD8^+^ T cells (**Figures 1E, F**), suggesting that complete absence of CD100 impairs the initiation of the early effector differentiation program. Additionally, CD100 deficiency resulted in a diminished capacity of CD8^+^ T cells to produce IFN-γ, although it didn’t affect Granzyme B production (**Figure 1G**; **Supplementary Figure 1D**). Collectively, these results indicate that CD100 knockout impairs the proliferation, activation, effector differentiation, and certain effector functions of HBcAg-specific CD8^+^ T cells.

### 3.2 mCD100 deficiency results in decreased proliferation and effector function of HBcAg-specific CD8^+^ T cells upon antigen stimulation *in vitro*

**Figure 2:**
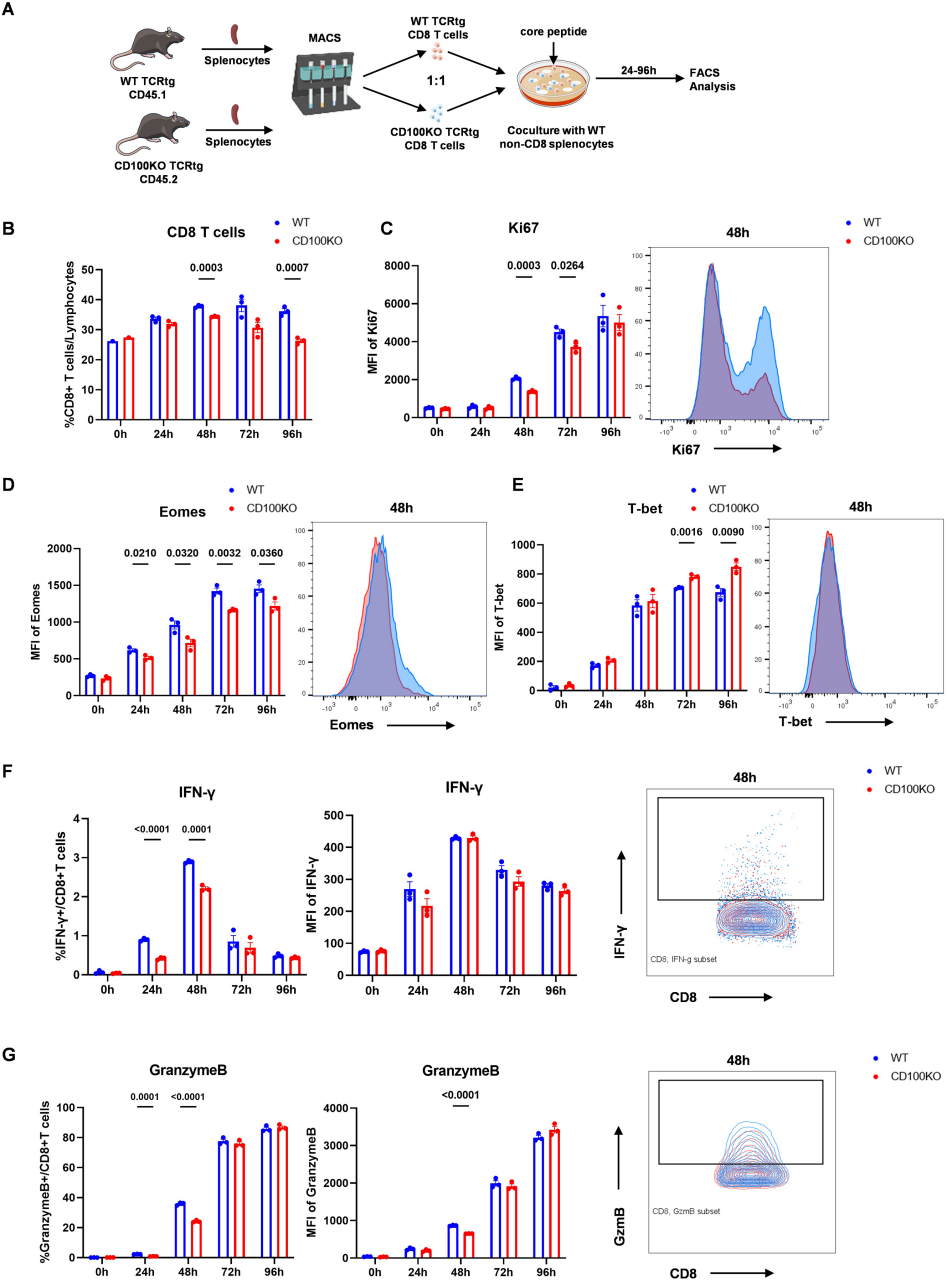
mCD100 deficiency results in decreased proliferation and effector function of HBcAg-specific CD8^+^ T cells upon antigen stimulation *in vitro*. **(A)** Experimental setup: CD8^+^ T cells were isolated from WT C93-TCR-transgenic and CD100KO C93-TCR-transgenic mice, mixed at a 1:1 ratio, and cocultured with WT non-CD8 splenocytes in the presence of Cor93 peptide for 96 hours. **(B)** Frequency of CD8^+^ T cells. **(C–E)** Ki67, Eomes and T-bet expression on CD8^+^ T cells were detected by flow cytometry. **(F–G)** Frequencies and mean fluorescence intensity (MFI) of IFN-γ and Granzyme B in CD8^+^ T cells are shown. Data are depicted as arithmetic means ± SEM. Differences between two groups were analyzed using unpaired Student’s t tests. MACS, Magnetically Activated Cell Sorting; KO, knockout.

In the preceding experiments, it remains unclear whether the observed effects on CD8^+^ T cell responses were mediated by the membrane-bound or the soluble form of CD100. To address this question, we separately isolated and purified CD8^+^ T cells from the spleens of WT and CD100KO C93-TCRtg mice. These cells were mixed at a 1:1 ratio and co-cultured with splenocytes depleted of CD8^+^ T cells from WT mice, in the presence of Cor93–100 peptide stimulation for 96 hours. Cells were collected at designated time points for flow cytometric analysis (**Figure 2A**; **Supplementary Figure 2A**). Consistent with our previous findings, the proportion of mCD100-deficient CD8^+^ T cells and their Ki67 expression intensity were significantly lower than those of WT CD8^+^ T cells (**Figures 2B, C**; **Supplementary Figure 2B**), indicating a markedly reduced proliferative capacity. However, no significant difference was detected in the expression level of the activation marker CD25 (**Supplementary Figure 2C**). Notably, the expression of Eomes was significantly reduced in mCD100-deficient CD8^+^ T cells, while T-bet expression showed an opposite trend (**Figures 2D, E**), which may stem from the dynamic balance and compensatory regulatory mechanisms between T-bet and Eomes during CD8^+^ T cell differentiation. Previous studies have suggested an inverse relationship between T-bet and Eomes expression (17, 18). The absence of mCD100 signaling may disrupt their homeostatic balance, skewing T cell differentiation toward a state that retains effector functions. Furthermore, during the early phase of antigen stimulation (within 48 hours), the proportions of mCD100-deficient CD8^+^ T cells producing IFN-γ and Granzyme B were both significantly decreased (**Figures 2F, G**). In summary, these data demonstrate that mCD100 acts cell-autonomously to sustain proliferation, effector differentiation and antiviral function of HBcAg-specific CD8^+^ T cells *in vitro*.

### 3.3 The function of mCD100 in promoting HBcAg-specific CD8^+^ T cell responses *in vivo*

**Figure 3:**
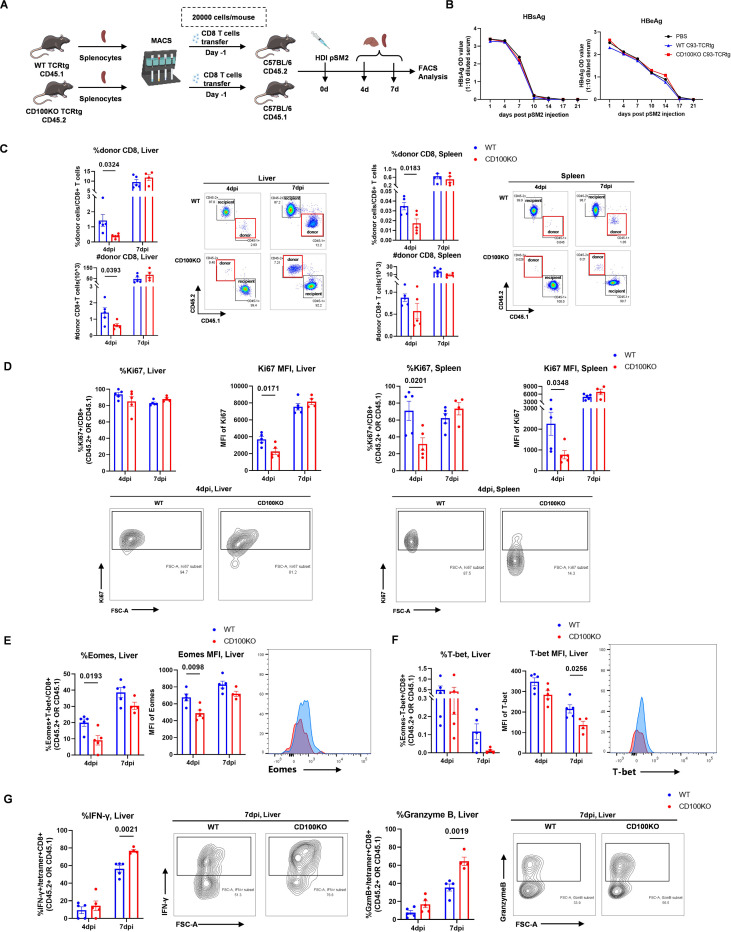
The role of mCD100 in promoting HBcAg-specific CD8^+^ T cell responses *in vivo.* **(A)** Experimental scheme: CD8^+^ T cells were isolated from WT C93-TCR-transgenic and CD100KO C93-TCR-transgenic mice, and 20,000 cells were adoptively transferred into wild-type C57BL/6 or C57BL/6.SJL recipient mice. The recipients were then hydrodynamically injected with the pSM2 plasmid after 1 day. Liver-infiltrating lymphocytes (LILs) and splenocytes were collected at 4 and 7 days post-injection (dpi) for analysis. **(B)** The kinetics of serum HBsAg and HBeAg were monitored. **(C)** Frequency and absolute number of donor CD8^+^ T cells in the liver (left) and spleen (right). **(D)** Frequency and MFI of Ki67 in donor CD8^+^ T cells from liver(left) and spleen(right). **(E, F)** Percentage and MFI of Eomes and T-bet in donor CD8^+^ T cells from the liver. **(G)** Proportion and MFI of IFN-γ and Granzyme B in donor CD8^+^ T cells from the liver. Five to six mice were analyzed per group, and at least two independent experiments were performed. Data are depicted as arithmetic means ± SEM. Differences between mouse groups were analyzed using unpaired Student’s t tests. HDI, hydrodynamic injection; dpi, days post-injection.

Next, we sought to further investigate how mCD100 directly regulates HBcAg-specific CD8^+^ T cell responses during the early phase post-viral exposure. We adoptively transferred 20,000 purified CD8^+^ T cells from either WT or CD100KO C93-TCRtg donors into naïve C57BL/6 recipients, followed one day later by hydrodynamic injection of pSM2 to establish an acute-resolving HBV replication model (**Figure 3A**). LILs and SPs were harvested at predetermined time points for flow-cytometric profiling of donor cells (**Supplementary Figure 3A**). Monitoring of viremia indicated that the transfer didn’t alter the viral clearance kinetics (**Figure 3B**). This excluded the possibility that viral replication levels confound T cell phenotype and function. At 4 days post-infection (dpi), both the frequency and absolute number of CD100KO donor CD8^+^ T cells were significantly reduced in liver (**Figures 3C**, left) and spleen (**Figures 3C**, right), demonstrating a significantly impaired expansion capacity of mCD100-deficient HBcAg-specific CD8^+^ T cells *in vivo*. Consistently, Ki-67 expression was markedly lower in CD100KO cells at 4 dpi (**Figure 3D**). Subsequently, we found that Eomes was diminished in CD100KO cells at 4 dpi, while a reduction in T-bet expression emerged at 7 dpi (**Figures 3E, F**). These findings are consistent with our *in vitro* observations with regard to the impaired early proliferation and altered effector differentiation of mCD100-deficient HBcAg-specific CD8^+^ T cells. Notably, although CD100KO donor cells showed increased IFN-γ and Granzyme B expression in the liver at 7 dpi (**Figure 3G**), this likely reflects a delayed compensatory rebound within the hepatic immune microenvironment rather than a genuine enhancement of T cell function. Because consistent trend was not observed in the spleen (**Supplementary Figures 3B, C**). Collectively, these results suggest that mCD100 expressed on resting HBcAg-specific CD8^+^ T cells operate as a co-stimulatory molecule, providing direct stimulatory signals for early T cell proliferation and effector differentiation.

### 3.4 Characterization of transcriptomic alterations in CD100KO HBcAg-specific CD8^+^ T cells

**Figure 4:**
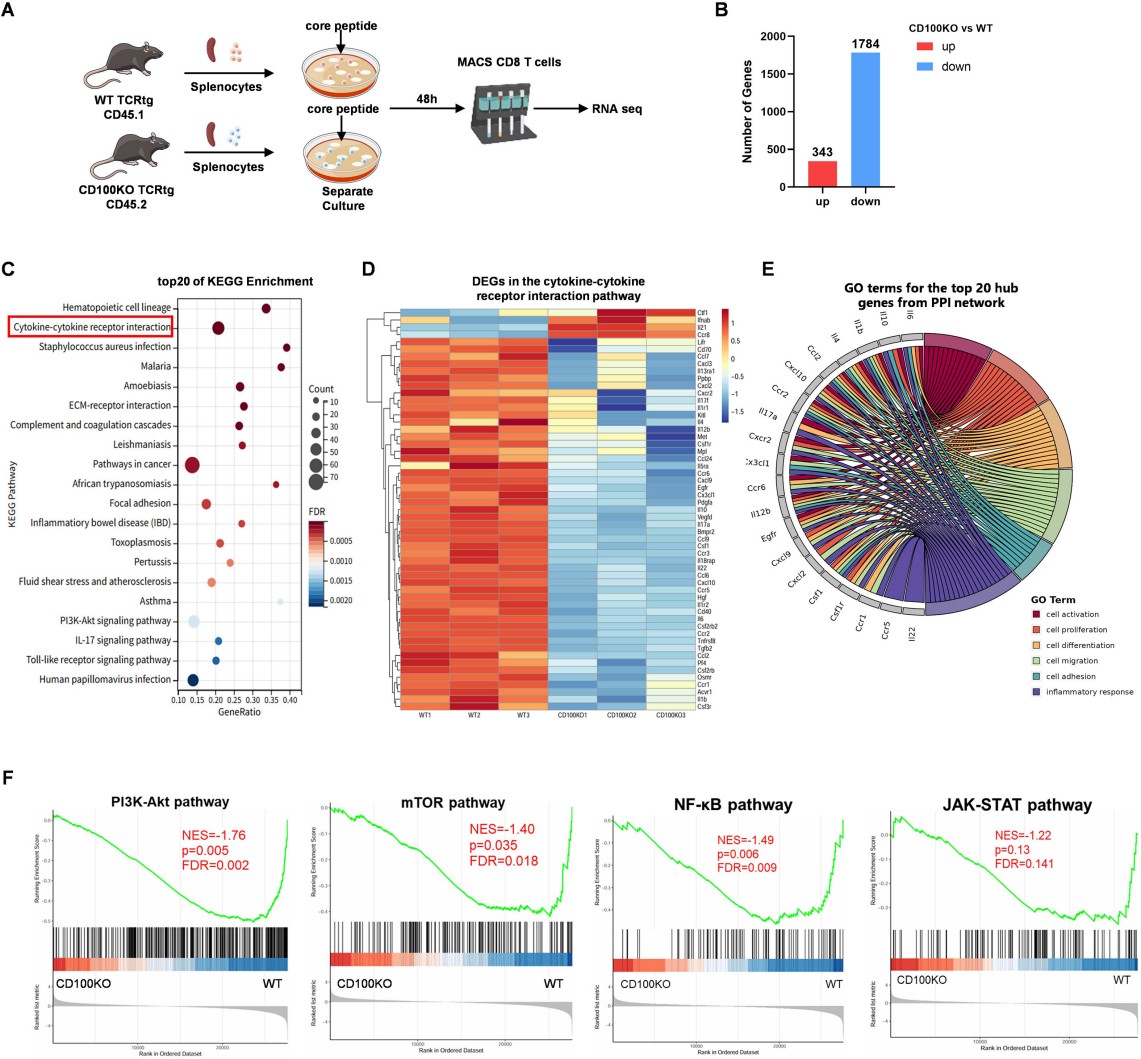
Characterization of transcriptomic alterations in CD100KO HBcAg-specific CD8^+^ T cells. **(A)** Experimental setup: Splenocytes from WT and CD100KO C93-TCR-transgenic mice were stimulated with Cor93 peptide for 48 hours, followed by CD8^+^ T cell isolation for RNA sequencing. **(B)** The number of DEGs between the two groups. **(C)** Top 20 KEGG enriched terms for significant regulated genes between two groups. **(D)** Heatmap depicting the expression levels of significant DEGs involved in the “cytokine-cytokine receptor interaction” KEGG pathway. **(E)** GO biological process terms for the top 20 hub genes (with highest connectivity) from the PPI network of DEGs in the “cytokine-cytokine receptor interaction” pathway. **(F)** GSEA reveals significant downregulation of gene sets associated with the PI3K-Akt, mTOR, NF-κB, and JAK-STAT signaling pathways in CD100KO CD8^+^ T cells. DEGs, differentially expressed genes; GSEA, gene set enrichment analysis; PPI, protein-protein interaction; FDR, false discovery rate; NES, normalized enrichment score.

To elucidate the mechanism by which mCD100 regulates CD8^+^ T cell responses, we conducted a comparative analysis of the transcriptomic profiles of WT and CD100KO CD8^+^ T cells. We harvested cells at the time point (48 hours post-stimulation) when phenotypic differences were most pronounced for RNA sequencing (**Figure 4A**). Differential expression analysis identified 1,784 genes significantly downregulated and 343 upregulated in CD100KO versus WT cells (**Figure 4B**). Notably, Prf1(encoding perforin) was downregulated and Lag3(encoding lymphocyte activation gene 3, an inhibitory checkpoint receptor) was upregulated in CD100KO CD8^+^ T cells (**Supplementary Figure 3D**), consistent with impaired effector function and possible accelerated exhaustion. KEGG pathway enrichment analysis indicated that these differentially expressed genes (DEGs) were significantly enriched in the “cytokine-cytokine receptor interaction” pathway, with the majority of genes within this pathway being markedly downregulated in CD100KO CD8^+^ T cells (**Figures 4C, D**). Protein-protein interaction network construction (**Supplementary Figure 3E**) followed by connectivity analysis highlighted a core set of 20 hub genes whose Gene Ontology (GO) annotations converged on cell activation, proliferation, differentiation, migration, adhesion and inflammatory response (**Figure 4E**). Furthermore, Gene Set Enrichment Analysis (GSEA) showed significant downregulation of gene sets associated with the PI3K-Akt, mTOR, NF-κB, and JAK-STAT signaling pathways in CD100KO CD8^+^ T cells (**Figure 4F**). Collectively, these results suggest that the promoting effect of mCD100 on the early proliferation and differentiation of HBcAg-specific CD8^+^ T cells observed in this study is likely mediated through the activation of PI3K-Akt-mTOR, JAK-STAT, and NF-κB signaling pathways.

## 4 Discussion

This study aimed to elucidate the direct regulatory role of mCD100 on HBcAg-specific CD8^+^ T cells and its underlying mechanism. Our findings demonstrate that both *in vitro* and *in vivo*, the absence of mCD100 expression leads to impaired proliferation, dysregulated effector differentiation (as indicated by altered expression levels of Eomes and T-bet), and changes in the production of effector molecules (IFN-γ and Granzyme B) in virus-specific CD8^+^ T cells during the early phase of antigen encounter. Transcriptomic analysis revealed that CD100 deficiency likely results in the broad downregulation of gene networks associated with cell activation, proliferation, and differentiation, potentially through the suppression of key signaling pathways including PI3K-Akt-mTOR, NF-κB, and JAK-STAT. These results strongly suggest that mCD100 is not merely a ligand acting on APCs or other immune cells, but can also function as a co-stimulatory molecule to directly participate in the regulation of T cell activation and differentiation.

Our finding aligns closely with the emerging concept of CD100’s “bidirectional signaling” (19). Conventionally, CD100 has been viewed primarily as an immunomodulatory ligand acting through its receptors CD72 and plexin-B1/B2 (20, 21). However, accumulating evidence suggests that membrane-bound CD100 itself can also function as a signaling receptor, mediating “outside-in” signal transduction (22, 23). Our study provides direct evidence for an intrinsic regulatory function of mCD100 within HBcAg-specific CD8^+^ T cells, filling a critical gap in understanding the role of CD100 in antiviral immunity.

It is noteworthy that in our *in vivo* experiments, despite impaired early expansion, mCD100-deficient cells produced more IFN-γ and Granzyme B at a later stage (day 7) in the liver. We do not interpret this as a true functional advantage caused by CD100 deficiency. Instead, we favor the explanation that this represents a delayed compensatory rebound secondary to impaired early priming and expansion. Because CD100KO cells undergo weaker initial activation, they may enter the effector phase later and be subjected to less early feedback inhibition, activation-induced restraint, or exhaustion-associated pressure in the hepatic environment. This phenomenon is time- and tissue-restricted, observed only in the liver at 7 dpi, not at 4 dpi nor in the spleen. Thus, we speculate this discrepancy may be attributed to the liver’s unique immune microenvironment and its complex regulatory networks.

Our transcriptomic analysis revealed that CD100 deficiency leads to significant downregulation of gene sets associated with multiple signaling pathways representing classic downstream events of T cell activation, including PI3K-Akt, mTOR, NF-κB and JAK-STAT (24). Therefore, we propose that mCD100 provides critical co-stimulatory signals and that these signaling pathways may be involved in mediating its effects. However, current transcriptomic data only support a correlative relationship, and direct causal evidence is still required.

Our findings uncover a dual therapeutic potential for targeting the CD100 axis in chronic HBV infection. As we previously demonstrated, exogenous sCD100 can break immune tolerance, reshape APC function, and indirectly enhance T cell responses (13). However, the present work suggests that direct intervention (such as agonistic agents) targeting mCD100 signaling could provide a more straightforward approach to deliver co-stimulation to exhausted HBcAg-specific CD8^+^ T cells, thereby promoting their proliferation and functional restoration.

Our study has several limitations that should be acknowledged. The hydrodynamic injection model does not fully recapitulate natural infection routes, and the adoptive transfer system bypasses physiological T cell priming. Therefore, validating these findings in patient samples or alternative infection models will be an important direction for future research.

In summary, this study further defines the role of mCD100 in anti-HBV immunity: it acts as an intrinsic co-stimulatory molecule on HBcAg-specific CD8^+^ T cells, directly providing critical signals for their early expansion, survival, and effector differentiation with transcriptomic evidence indicating potential involvement of key signaling pathways including PI3K-Akt-mTOR, NF-κB, and JAK-STAT. These findings provide novel insights and potential targets for developing new immunotherapeutic strategies against HBV and other viral infections.

## Data Availability

The datasets presented in this study can be found in online repositories. The names of the repository/repositories and accession number(s) can be found below: https://www.ncbi.nlm.nih.gov/bioproject/PRJNA1432310.
