# Supplementary material for: Cell-autonomous co-stimulatory function of membrane-bound CD100 promotes activation and differentiation of HBcAg-specific CD8+ T cells
Source: Front Immunol. 2026 Jun 3;17:1826036. doi: 10.3389/fimmu.2026.1826036 (PMC13272529; doi:10.3389/fimmu.2026.1826036)
Supplement: Supplementary file 1 [file Table1.docx]

| **Mouse antibodies-Flow cytometry** | |  |  |  |  |
| --- | --- | --- | --- | --- | --- |
| **Antibody** | **Fluorophore** | **Clone** | **Company** | **Catalog #** | **Dilution** |
| **CD45.1** | **FITC** | **A20** | **Biolegend** | **110706** | **1:200** |
| **CD45.1** | **PE** | **A20** | **Biolegend** | **110708** | **1:200** |
| **CD45.1** | **PerCP-Cy5.5** | **A20** | **BD** | **560580** | **1:200** |
| **CD45.2** | **PE-Cy7** | **104** | **Biolegend** | **109830** | **1:200** |
| **CD45.2** | **BV421** | **104** | **BD** | **562895** | **1:200** |
| **CD3** | **PE** | **145-2C11** | **BD** | **553063** | **1:200** |
| **CD8** | **APC-Cy7** | **53-6.7** | **BD** | **557654** | **1:200** |
| **CD100** | **PE** | **BMA-12** | **Biolegend** | **147604** | **1:200** |
| **CD25** | **BB700** | **PC61** | **BD** | **566498** | **1:200** |
| **Eomes** | **PE-Cy7** | **Dan11mag** | **Invitrogen** | **25-4875-82** | **1:100** |
| **T-bet** | **PerCP-Cy5.5** | **O4-46** | **BD** | **561316** | **1:200** |
| **Granzyme B** | **PE** | **QA16A02** | **Biolegend** | **372208** | **1:200** |
| **Ki67** | **BV421** | **16A8** | **Biolegend** | **652411** | **1:100** |
| **IL-2** | **PerCP-Cy5.5** | **JES6-5H4** | **Biolegend** | **503822** | **1:200** |
| **IFN-γ** | **APC** | **XMG1.2** | **Biolegend** | **505810** | **1:200** |
| **TNF-α** | **FITC** | **MP6-XT22** | **Biolegend** | **506304** | **1:200** |
| **H-2K^b^ HBV core Tetramer** | **APC** | **MGLKFRQL** | **MBL** | **TS-M537-2** | **1:100** |

**Supplementary Table 1**. List of flow cytometry antibodies used for staining.


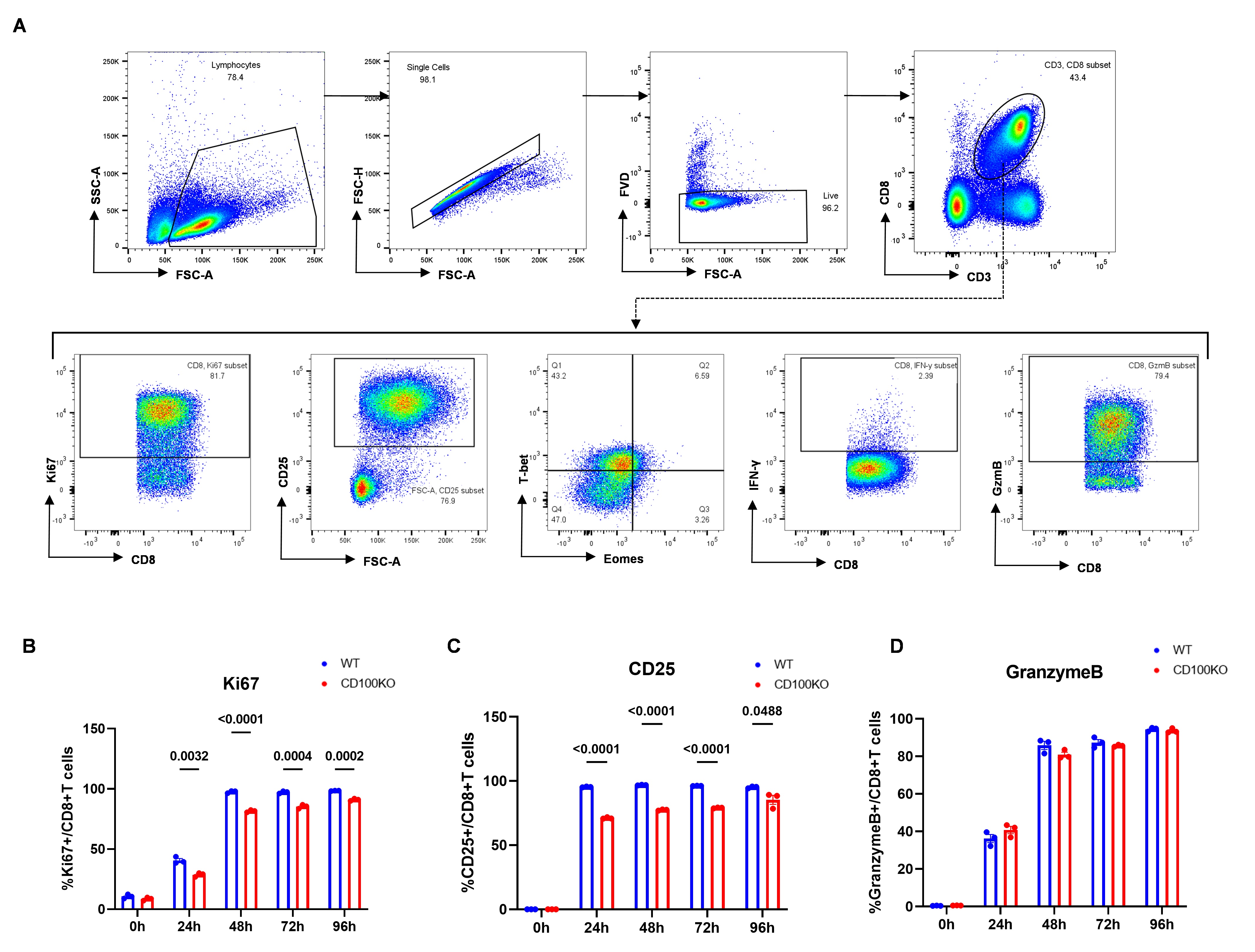


**FigureS1. Related to Figure 1.** (A) Gating strategies for FACS analysis of CD8^+^ T cell function. Frequencies of Ki67(B), CD25(C) and Granzyme B(D) in CD8⁺ T cells are displayed. Data are depicted as arithmetic means ± SEM. Differences between two groups were analyzed using unpaired Student’s t tests.


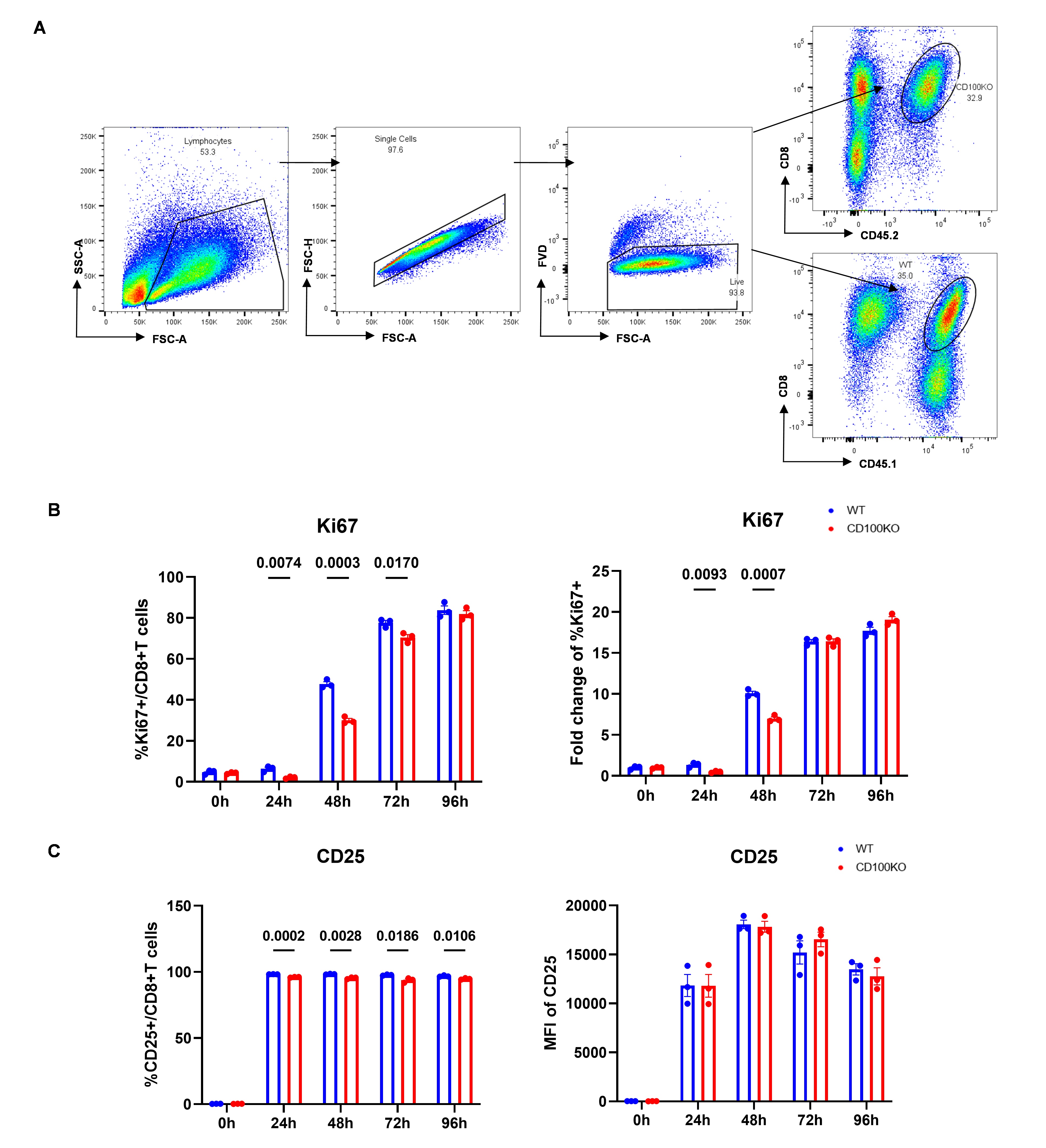


**FigureS2. Related to Figure 2.** (A) Gating strategy for identifying WT and CD100KO CD8⁺ T cells from lymphocytes. (B) Frequencies of Ki67 and fold change of frequencies in CD8⁺ T cells are displayed. (C) Frequencies and mean fluorescence intensity (MFI) of CD25 in CD8⁺ T cells are shown. Data are depicted as arithmetic means ± SEM. Differences between two groups were analyzed using unpaired Student’s t tests.


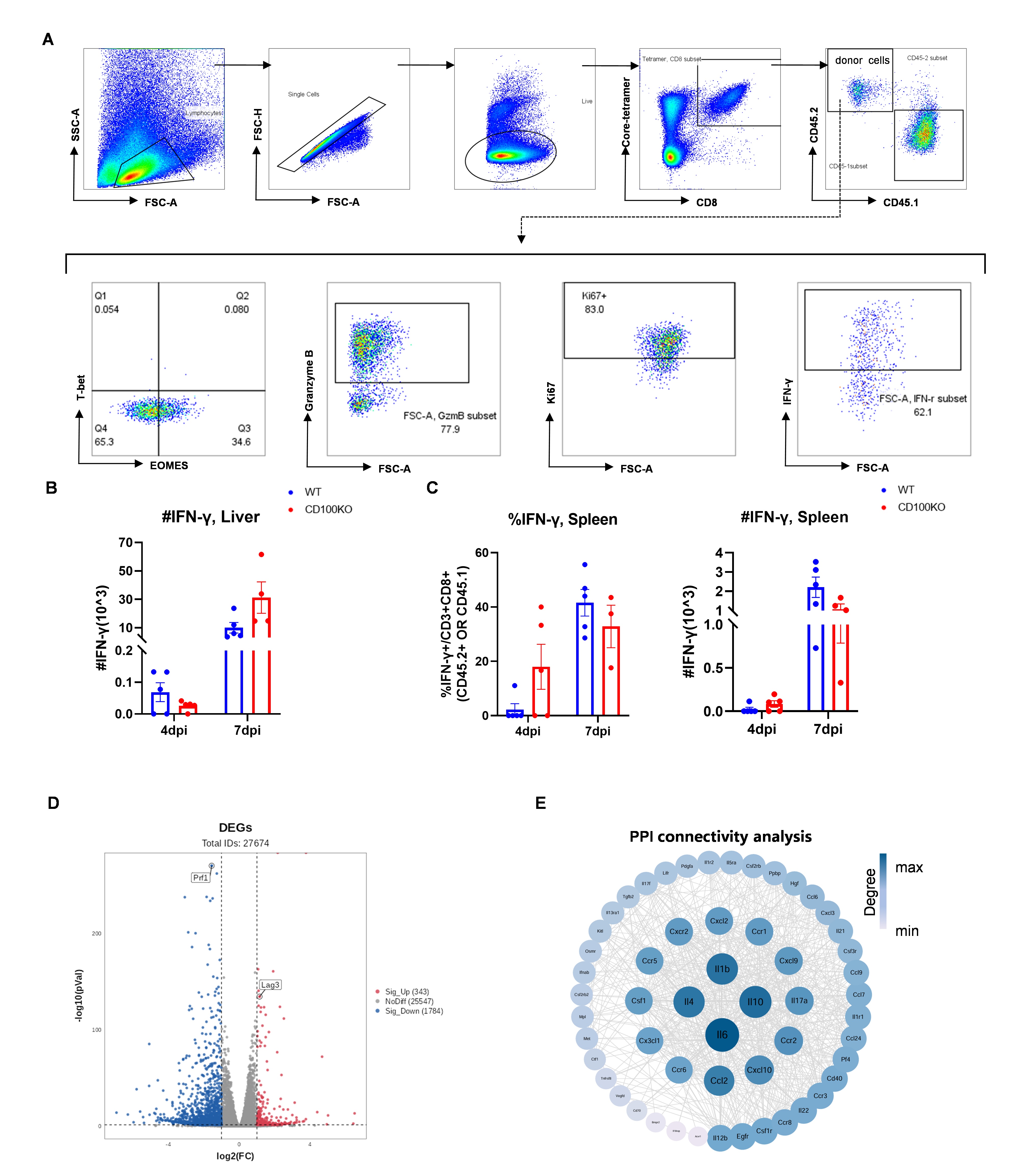


**FigureS3. Related to Figure 3. and Figure 4.** (A) Gating strategy for identifying donor CD8⁺ T cells from lymphocytes. (B) Absolute number of IFN-γ-producing donor CD8⁺ T cells in the liver. (C) Frequency and absolute number of IFN-γ-producing donor CD8⁺ T cells in the spleen. (D) Volcano plots showed differentially expressed genes between two groups. (E) Construction of a PPI network for differentially expressed genes (DEGs) from the "cytokine-cytokine receptor interaction" pathway. PPI, protein-protein interaction.
